# Quality of Life in Patients Undergoing Percutaneous Transluminal Coronary Angioplasty (PTCA)

**DOI:** 10.5539/gjhs.v7n5p246

**Published:** 2015-03-16

**Authors:** Fatemeh Bahramnezhad, Mahboobeh Khajeh, Mahmoud Shiri, Parvaneh Asgari, Pouya Farokhnezhad Afshar

**Affiliations:** 1 Nursing and Midwifery Faculty, Tehran University of Medical Sciences, Tehran, Iran; 2 Electronic engineering, Islamic Azad University Iranshahar Branch, Iranshahar, Iran; 3 Nursing and Midwifery Faculty, Arak University of Medical Sciences, Arak, Iran; 4 Nursing and Midwifery Faculty, University of Social Welfare and Rehabilitation, Tehran, Iran

**Keywords:** Quality of life, percutaneous, Transluminal Coronary Angioplasty (PTCA), follow-up

## Abstract

**Background:**

Coronary artery diseases are the main causes of death in industrial Countries. Transluminal angioplasty is a common technique used to manage the condition of coronary arteries. The purpose of this study was to explore the quality of life in patients sustaining this measure in two stages before the procedure and then three consecutive after that 3, 6 and 12 months respectively.

**Materials and Methods:**

This research was a longitudinal study and data was collected between 2011-2013 years. 115 patients were included. Data were collected through using a questionnaire with 40 questions. The subjects before, 3, 6 and 12 months after the procedure filled out questioner. Data were analyzed by statistical tests including T- test, Fisher exact test, Wilcoxon and Friedman with Software SPSS version 16, P value<0.05.

**Results:**

There were significant differences in the quality of life in patients with PTCA before procedure and 3 months after that (P=0.004). Quality of life of patients undergoing PTCA in the four levels, three, six and twelve months after the operation had a significant difference (P <0.001).

**Conclusions:**

Quality of life of people with PTCA operation three months after surgery is reduced. It is required during this period the patient treatment team and supports his family and put under the necessary training in this period to give patients and encourage them to pursue their condition should.

## 1. Introduction

As one of the most common medical problems and the most important reason of death around the world are coronary artery diseases (Rahmani & Mollashahi, 2013). If the medical treatment is unsuccessful, PTCA is considered as an efficient treatment measures in this situation (Barakate, Hemli, Hughes, Bannon, & Horton, 2003). In 1999 it was estimated that 1 million PTCA in the USA have been conducted. Following PTCA patients usually spend only one single day in hospitals, so this method is more economical than the other invasive measurements such as coronary artery bypass graft (CABG) (Echteld, Vanelderen, & Leo, 2001). However, patients sustaining PTCA should continue their follow-up visits with the hospitals or clinics over the next 5 years (Serruys et al., 2001). Illness by itself can cause disability for the patients, so may alter the Quality of life (QOL) (physical & psychological (van Dijk et al., 2000). QOL is a multifaceted concept including physical, Psychological and social aspects. In other hands, it is well being resulting from satisfaction or dissatisfaction of various aspects of life which are important to a given person (Bahramnezhad Noughabi, Afshar, & Marandi, 2013).

QOL is considered as a significant outcome of being affected by a disease or the patient’s response to a particular treatment or a special process (Eastwood, Doering, Dracup, Evangelista, & Hays, 2011).

Over the last two decades a great deal of attention has been paired to assessing and improving the quality of life especially for chronically ill clients. Making improvement in daily function and quality of life in chronic conditions are addressed as a valuable goal. During the last ten years, many studies have been done to explore the quality of life in patients with cardiac disease and they proved in this area (Kappetein et al., 2011). The presence or absence of the necessity for making any change in lifestyle is an improvement factor in quality of life after sustaining PTCA (Pasquali Alexander, Coombs, Lytli, & Peterson, 2003). QOL is not related to the time after procedure but it is associated with some other factors such as gender (lower level of QOL in women after PTCA), age, unstable angina, diabetes mellitus, and multi veins involvement and Thrombosis formulation (Fortescue Kahn, & Bates, 2003). Patients with PTCA Complain of the high level of weakness and restriction in there and more anxious compared to those who experienced CABG (Hueb et al., 2010). The sexual satisfaction of the patients improves until 8 years after PTCA. As a result, their quality of life improved (H. Lukkarinen & O. Lukkarinen, 2007). Regarding the importance of PTCA in treatment patients with coronary artery disease and it’s complications in patients, and also considering the importance of quality of life in patients with PTCA, this study has been designed to determine the QOL before PTCA three, six and twelve months after admission in Tehran University’s hospitals.

## 2. Materials and Methods

This study was a longitudinal study and the data was collected between 2011-2013 years.

The study population is patients referred to hospitals of Tehran University of Medical Sciences. Since this study was designed for a period of one year, it was predicted that many subjects might leave the study; therefore, this group was established by 115 subjects but in the end remained 64 patients. The criteria for inclusion in our study were included; age less than 65, not history of chronic mental or physical illnesses, literacy or living with a literate person, access to telephones and not being dependent on the pacemaker. Exclusion criteria were the need for pacemaker and Coronary artery bypass graft during the study. Data were collected through using a questionnaire with 40 question including 6 demographic questions, 5 questions about the present condition and 29 questions on the QOL (Quality of life tool). Interview and self-reporting were applied to answer to these questionnaires. This instrument has been used in many studies and its validity and reliability is proved. The title and objectives of the study were explained to each subject and they were informed that they would be able to leave the study whenever they prefer and it is not related to their relationship with the health care team or physician. The subjects before and 3, 6 and 12 months after the procedure filled out questionnaires. Another questionnaire along with an instruction explaining how to fill if he had not answered the questionnaire by one month and despite the telephone reminding. The phone number of the researcher was available for subjects and they were allowed to call and ask questions or consult in case of any problem. They also were informed that if they like to make an appointment to see the physician, the researcher would help them. Data were analyzed by statistical tests including T- test, Fisher exact test, Wilcoxon and Friedman with Software SPSS version 16, P value<0.05. The informed consent was received from the patients after fully explaining all the cases mentioned in the form for the patients and signing the form in case of being willing to participate in the study.

## 3. Results

According to the findings, the average age of the patients with PTCA was 51.65. Demographic characters of the samples are displayed in Table 1.

**Table 1:** Demographic characters of the respondent in group

Demographic		N (%)
Age(years)	40>	10(8/7)
	40-50	41(35/7)
	50-60	62(35/9)
	60>	2(1/7)
Marriage	Single	2(1/7)
	Married	113(98/3)
Time of diagnosis (month)	**<1**	52(45/2)
	**1-6**	47(40/9)
	**6-12**	10(8/7)
	12-24	2(1/7)
	**24<**	4(3/5)

According to the information, there were significant differences in the QOL in patients with PTCA before procedure and 3 months after that. In other hands, the average of the score of QOL decreased 3 months after PTCA compared to the score before conducting the procedure (P=0. 04). The findings showed that the majority of the patients had a moderate level of QOL before PTCA, which improved and changed into desirable 6 months after PTCA. There was also a significant difference between the QOL before PTCA and 6 months after that. In fact 6 months after PTCA, the QOL improved compared before that. (P<0.001) (Table 2).

**Table 2:** Distribution of QOL in patients undergoing PTCA relative frequency before and three, six and twelve months after surgery

Time QOL	Before	3months	6Months	12Months	Testing
	N(%)	N(%)	N(%)	N(%)	
Unfavorable	25(21/7)	40(48/2)	10(13/9)	22(34/4)	Friedman test x^2^=153/709 df=3 P<0.001
Desirable	75(65/2)	32(38/6)	23(31/9)	34(53/1)	
Favorable	15(13)	11(13/2)	39(54/2)	8(12/5)	
Total	115(100)	83(100)	72(100)	54(100)	
Mean	61/348	58/087	64/478	50/53	
SD	16/160	27/342	20/659	18/532	

## 4. Discussion

Findings showed that there was not a significant difference in the QOL in patients with PTCA before the procedures and 3 months after enduring them.

Without on effective therapy, on ischemic heart disease can lead to an Intermittent angina, a drop in the blood supply, dysrhythmia and a decrease in physical activities which make a patient incompetent for his work and other psychosocial problems (Choi, Hwang, Lee, & Kim, 2009). According to the results of the study 6 months after PTCA and CABG the QOL was higher for those with PTCA. Rue et al announced that the sooner discharge from the hospital the higher QOL (Ruo et al., 2003). El Baz et al are of the opinion that *“the main reason for decreasing in QOL first months after PTCA is remaining physical signs and symptoms of the condition specially fatigue. Moreover, the QOL will improve if the patient is provided with psychosocial support and encouragement for follow-up care during one year after PTCA*” (El Baz, Middle, Van Dijk, Boonstra, & Reijnevled, 2009). The results of the above study reinforce the findings of the present study. The QOL decreases by 3 months of surgery, but after 6 and 12 a month increase. Mercier suggests the QOL doesn’t change in patients with PTCA owing to the risk of repeating vascular obstruction and consequently repeating the procedure, besides if there is another condition such as Diabetes at the same time, the QOL will lessen even more (Mercier, 2008). These results do not verify the finding of the present study. According to the finding of our study, the QOL diminished 3 months after PTCA, but it showed a rise 6 and 12 months after that. Brosson believe that the QOL is at a low level only 1 to 2 weeks after PTCA and this interval is the time in which the patients are still suffering from chest pain, fatigue and weakness and shortness of Breath. Relieving of these symptoms means a growth in patient’s QOL. They also argue that the QOL will not decrease even if the patient has to endure another PTCA (Brorsson et al., 2002). Mastright claims that regardless of any change in the physical symptoms, the QOL will improve after PTCA and this is due to the psychological support given to the patients after Procedure. They also assume that the QOL will grow initially because of the patients, belief and trust on the positive results of the Procedure; however, it may decrease over time if the patient does not experience any improvement in his condition (van Mastrigt et al., 2010). These results are not in harmony with the findings of the present study. According to our findings in this study, the QOL raises immediately after the procedure. Researchers believe that the possible cause of a decline in the level of QOL during the first 3 months after PTCA is remaining sign and symptoms that the patients expected to be subsiding. Boulliera et al argue that 40%-50% of the patients with the PTCA need to repeat the procedure by 6 months of the first try and this factor affect the QOL (Boullier et al., 2000). Echterld believes that there is a growth in the QOL over 4-6 weeks after PTCA but over a longer period owing to the need for repeating the procedure it may show a fall (Echteld, Vanelderen, & Leo, 2001). Fortescue suggests that both PTCA and CABG lead to relieve angina and restore working ability in patients; but the point is that CABG is more effective in fall down angina while PTCA is faster in its effect on the patient’s ability to resume his job. After several years, the general outcome of both methods is similar (Fortescue, Kahn, & Bates, 2003). Since the PTCA is a common procedure for coronary artery disease around the world, the researcher recommends, factors that influence the quality of life in patients with PTCA investigated. Since, this study was the need for follow up, limitation had such as a change of address and telephone number of patients, lack or failure of a questionnaire or died in some cases. Also, lack of control over the factors affecting quality of life was a limitation.

## 5. Conclusion

Quality of life of people with PTCA operation three months after surgery is reduced. It is required during this period the patient treatment team and supports his family and put under the necessary training in this period to give patients and encourage them to pursue their condition should.
